# Fully automated breast segmentation on spiral breast computed tomography images

**DOI:** 10.1002/acm2.13726

**Published:** 2022-08-09

**Authors:** Sojin Shim, Davide Cester, Lisa Ruby, Christian Bluethgen, Magda Marcon, Nicole Berger, Jan Unkelbach, Andreas Boss

**Affiliations:** ^1^ Institute of Diagnostic and Interventional Radiology University Hospital of Zurich Zurich Switzerland; ^2^ Department of Radiation Oncology University Hospital of Zurich Zurich Switzerland

**Keywords:** breast, CT, density, segmentation

## Abstract

**Introduction:**

The quantification of the amount of the glandular tissue and breast density is important to assess breast cancer risk. Novel photon‐counting breast computed tomography (CT) technology has the potential to quantify them. For accurate analysis, a dedicated method to segment the breast components—the adipose and glandular tissue, skin, pectoralis muscle, skinfold section, rib, and implant—is required. We propose a fully automated breast segmentation method for breast CT images.

**Methods:**

The framework consists of four parts: (1) investigate, (2) segment the components excluding adipose and glandular tissue, (3) assess the breast density, and (4) iteratively segment the glandular tissue according to the estimated density. For the method, adapted seeded watershed and region growing algorithm were dedicatedly developed for the breast CT images and optimized on 68 breast images. The segmentation performance was qualitatively (five‐point Likert scale) and quantitatively (Dice similarity coefficient [DSC] and difference coefficient [DC]) demonstrated according to human reading by experienced radiologists.

**Results:**

The performance evaluation on each component and overall segmentation for 17 breast CT images resulted in DSCs ranging 0.90–0.97 and in DCs 0.01–0.08. The readers rated 4.5–4.8 (5 highest score) with an excellent inter‐reader agreement. The breast density varied by 3.7%–7.1% when including mis‐segmented muscle or skin.

**Conclusion:**

The automatic segmentation results coincided with the human expert's reading. The accurate segmentation is important to avoid the significant bias in breast density analysis. Our method enables accurate quantification of the breast density and amount of the glandular tissue that is directly related to breast cancer risk.

## 1 INTRODUCTION

Breast cancer constitutes more than a quarter of cancer occurrences among women and is the second cancer most frequently leading to a woman's death.^1^ In order to reduce the mortality rate by early cancer diagnosis and prevention of late stage breast cancer development, breast imaging technologies have been developed, and studies for assessing the breast cancer risk based on those images have been conducted.

Previous studies widely observed a strong association of breast density with increased breast cancer risk. Breast density affects the risk of developing breast cancer in two different ways. First, the quantitative density of the breast is directly interrelated to the breast cancer: According to a recent study, the odds ratio for developing breast cancer for the most dense compared with the least dense categories ranged from 1.8 to 6.0.^2^, ^3^, ^4^ Second, dense breast tissue decreases the sensitivity of mammograms by masking cancerous tissue.^5^

Although medical imagings, such as mammography, ultrasonography (US), or breast magnetic resonance imaging (MRI), rely on potentially quantifiable measures of physical properties, such as cumulative attenuation or reflection of the projected beams or polarized atomic spin's relaxation time, the images presented to the reader are usually gray level images representing the relative contrast of the signal. The contrast may be nonlinear and may also be further distorted by additional signal processing. Without additional tools, the breast imagers can only roughly estimate the breast density. For mammography and US, the breast density is only visually assessed following the Breast Imaging Reporting and Data System (BI‐RADS)’s classification by the American College of Radiology.^4^ On breast MR images, Yaffe,^6^ Wu,^7^ Gubern‐Merida,^8^ and Dalmış ^9^ computationally estimated the breast density by calculating the ratio of the number of voxels classified as the glandular and adipose tissues after segmenting the breast image based on the imaging signal intensity. However, their density estimation solely relays on the segmented two‐class binary map comprising voxels in 0.5–1.6‐mm width assuming the image has only two discrete true gray levels and ignoring the continuous gray level values partially due to the partial volume effect in voxels. Therefore, the estimated breast density by their methods might substantially vary depending on the segmentation algorithm applied as seen in the variation of the estimated density of up to 10% in Ref.^9^

Spiral breast computed tomography (CT) equipped with a photon‐counting detector technology enables the assessment of the quantitative density of each voxel of the breast CT data.^10^ The image of breast CT presents each voxel as the radiological density of the tissue located in the corresponding voxel, expressed in Hounsfield units (HUs). Despite the potential for the quantitative assessment of breast density by means of the breast CT, however, radiologists, due to the lack of a suitable quantitative procedure, have so far only qualitatively examined the images.

Segmentation of breast CT images is necessary to properly assess the quantitative breast density. In addition, a segmentation procedure can further benefit the diagnostic process and the quality control in several ways:

1. It enables the automatic assessment of the individual breast density.
2. It enables the calculation of the breast organ dose of individuals and the dose coefficient for general dose assessment in clinics by applying a Monte Carlo radiation dose simulator.
3. The precise localization of the pectoral muscle and skin may allow the automatic analysis of the position of the breast and automatize quality control in spiral breast CT examinations.
4. Breast population studies for several breast features (e.g., distribution of glandular tissue, amounts of glandular tissue and skin, and presence of pectoralis muscle and rib) can be automatically conducted.

**FIGURE 1:**
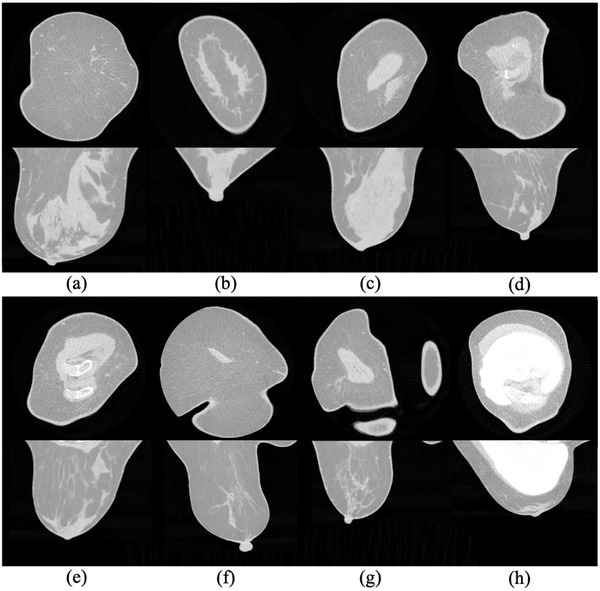
Eight exemplary spiral breast computed tomography (CT) images in coronal (top) and transverse or sagittal (bottom) planes

Breast images can feature substantially varying structures and shapes as depicted in the coronal CT images of breasts in Figure 1; some breast components, such as the rib, pectoralis major muscle, additional skinfolds from the thoracic or abdominal wall depicted by the breast CT, and silicone implant, may appear in some images but not in others. Their spatially varying appearance caused by both nature and imaging artifacts and the neighboring distracting structures present a challenge for the locating and segmenting process. Further difficulties lie in the physical connection of the glandular tissue to the skin and pectoralis muscle, which makes the separation of the tissues vague, the overlapping HU distribution among the soft tissues making investigation and separation among the nearby soft tissues difficult, and the resolution degradation inherent in the imaging system leading to the blurring of the components’ image boundary.

We implemented a fully automated algorithm to perform the image segmentation and analysis on the individual breast CT images. A pipeline of dedicated algorithms was implemented to segment all possible breast components regarding the previously mentioned obstacles; the complete framework is defined by the sequence of these algorithms. The method did not require a time‐consuming training on a large number of manual labels by specialized human readers. The performance of our breast segmentation algorithm on the spiral breast CT images was evaluated by computing the spatial overlap evaluation metrics for image segmentation in comparison to manual segmentation by two radiologists as a reference and by a five‐point Likert scale test conducted by five experienced radiologists.

## 2 METHODS

### 2.1 3D breast image by a novel breast CT with photon‐counting detector (PCD) technology

All images presented in this paper are acquired with a spiral breast CT system (nu:view, AB‐CT—Advanced Breast‐CT GmbH, Erlangen, Germany) equipped with a CdTe PCD (Direct Conversion, Danderyd, Sweden) applying an optimized image acquisition setup.^9^, ^10^ All spiral breast CT images used in this study are reconstructed at 300 μm × 300 μm × 300 μm voxel size by applying a soft kernel (Shepp–Logan) and a Feldkamp‐type filtered back projection algorithm.^11^ Each breast CT image consists of 682 × 682 × (300–900) voxels with the sagittal axis’ length depending on the length of the breasts. The HU values were reliable across a variety of conditions such as breast size or implant presence except the area where artifacts appear.^12^, ^13^, ^14^

### 2.2 Dataset

The segmentation method was aimed to segment the following soft tissues (1–5) and strongly absorbing materials (6 and 7):

1. adipose tissue
2. glandular tissue
3. skin
4. pectoralis major muscle
5. skinfolds from the thoracic or abdominal wall
6. ribs
7. silicone implants

The method was developed on healthy breast CT images from 68 preselected patients. Not all of these tissues are present in every image. Every image contains the skin with different thicknesses and the glandular and adipose tissues in different proportions as an example shown in Figure 1a,b. Seventeen of the preselected datasets partly comprised the pectoralis major muscle, and five among the breast CT scans exhibited rib structures (e.g., Figure 1d,e). Folded skin sections were included in the seven CT scans (Figure 1f,g). Ten breasts had a silicone implant (Figure 1h). Only one breast per patient was included in the preselection in order to diversify the samples and avoid redundancy in the analysis.

For the evaluation of the segmentation algorithm, 25 representative breast CT images were selected among the ones who were not preselected in the development set. Five breast images were chosen to represent each BI‐RADS's mammographic density class: almost entirely fatty, scattered fibroglandular density, heterogeneously dense, extremely dense. In addition, five breast CT images of implanted breasts were included in the test set. For the manual segmentation of each of skin, pectoralis muscle, skinfold section, and silicone implant, five breast CTs, including the corresponding objects, were selected within the evaluation set.

### 2.3 Preliminary study on the HU values of the breast components in the spiral breast CT image

In order to adapt the HU values for the segmentation and the breast density analysis, we analyzed the HU distributions of the components on in vivo breast CT images. We acquired the reference HU values following three steps: acquisition of the reference segmentation of human reading for the components except the adipose and glandular tissues, histogram analysis to separate the components that were not segmented, and analysis of the HU values of each component.

#### 2.3.1 Reference manual segmentation by radiologists

**FIGURE 2:**
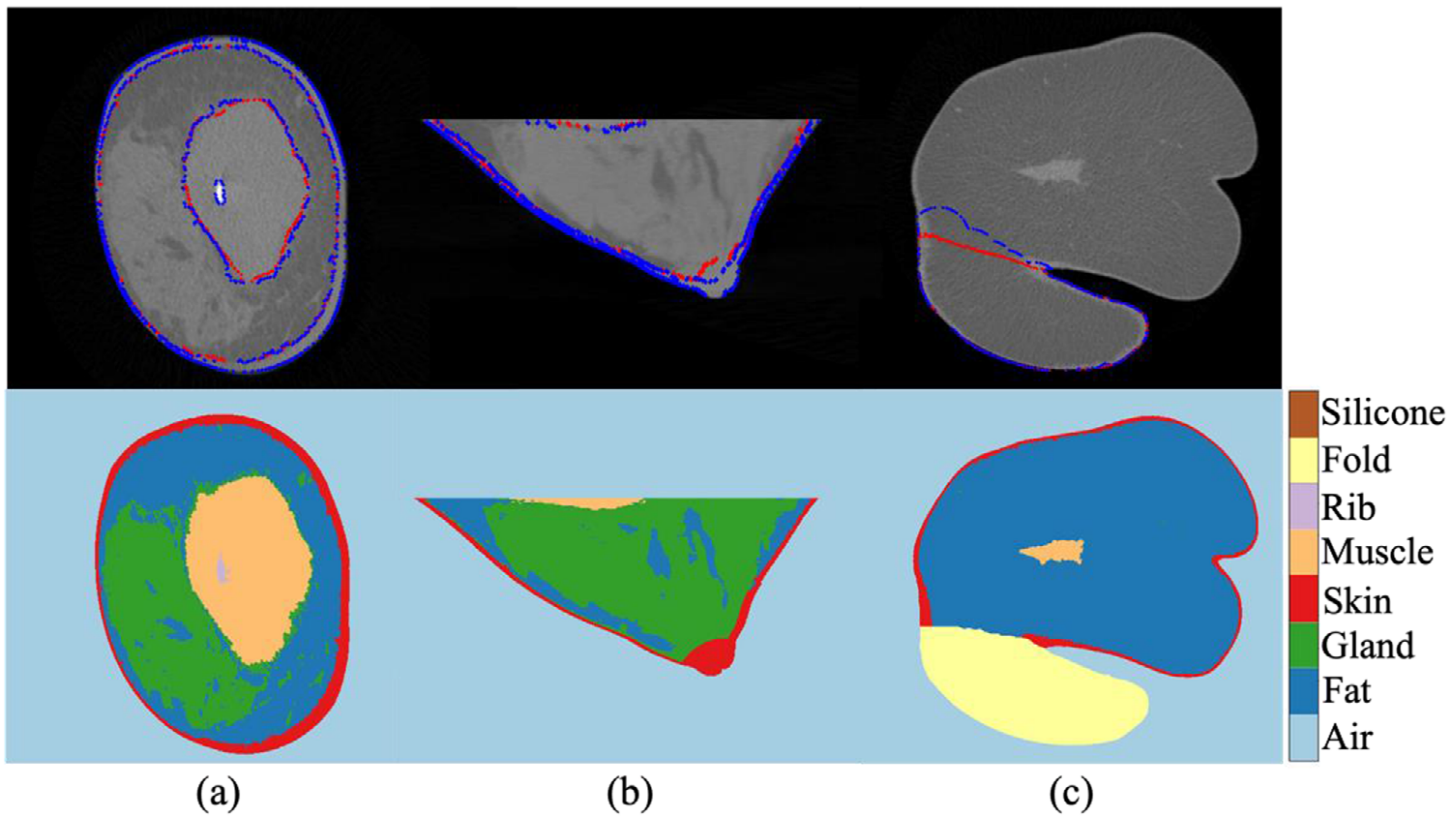
Breast component segmentation by readers (top) and our automatic segmentation algorithm (bottom) for the pectoralis muscle and skin (a) and (b) and skinfold section (c)

Third‐ and fourth‐year radiology residents, referred to as the readers A and B in the results, manually segmented all the five tissues listed in Section 2.2 except the glandular and adipose tissues—the skin, pectoralis muscle, skinfold section, ribs, and silicone implants (see Figure 2). Due to the complex structure of the glandular and adipose tissues and the mixed HU values of the two tissues caused by the partial volume effect in a large number of voxels, which may lead to a significant inherent uncertainty in the manual segmentation, no manual segmentation of these breast soft tissue components could be conducted. In the manual segmentation process, the radiologists used one medical image analysis software, MIM Maestro (MIM Software Inc., Belgium). The radiologists individually painted each component slice by slice using a 2D brush with the aid of a thresholding mask. The masking threshold was selected by comparing the masked edge to the object's visual edge on the image by each radiologist. The reproducibility of the manual segmentation was assessed by volume overlap analysis between the two manually segmented image matrices by the two radiology residents.

#### 2.3.2 Histogram processing on HU values of the adipose and glandular tissues

**FIGURE 3:**
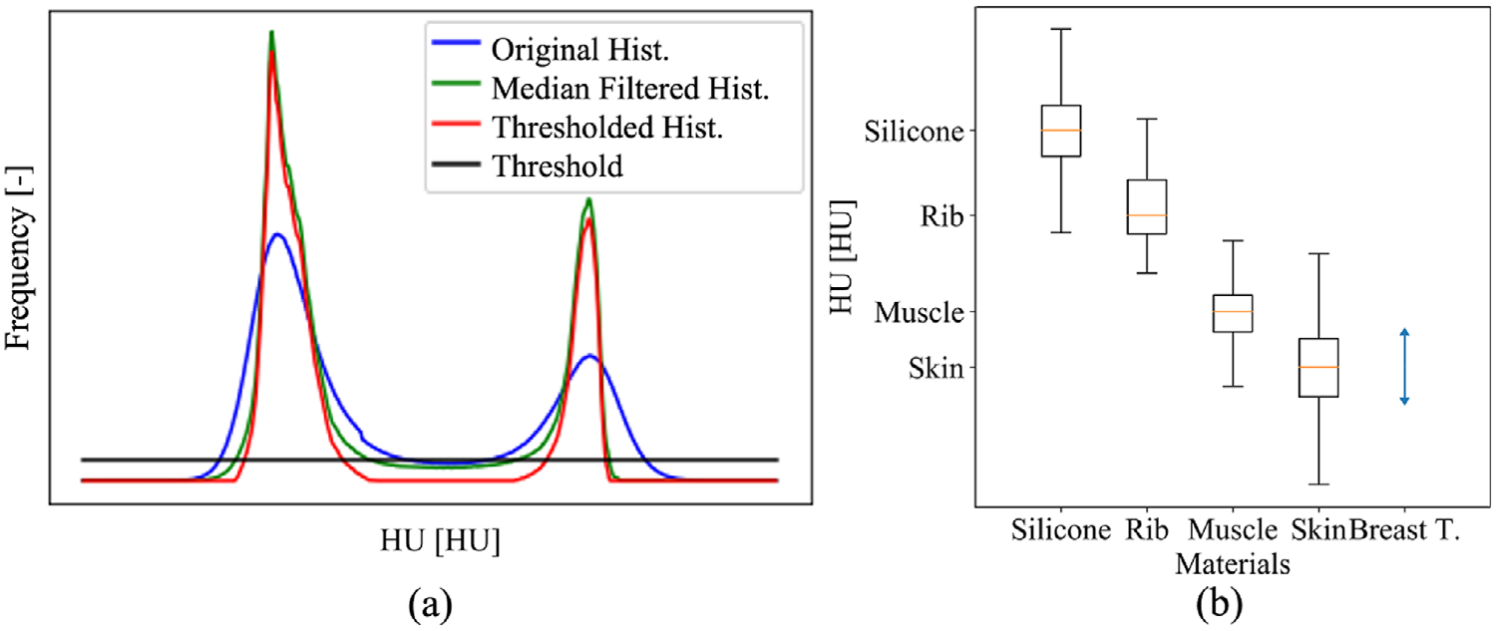
Schematic Hounsfield unit (HU) distribution of the adipose and glandular tissue in a real breast computed tomography (CT) image (a) and box‐and‐whisker diagram for the HU distribution of the breast components (b). In (b), the boxes represent the IQR, the red lines the median, and the blue error bar 95% HU range.

The breast tissue image data separated from the manually segmented components in Section 2.3.1 exhibits mixed HU values of glandular and adipose tissues mainly due to partial volume effects. The mixed signals shape the histogram as shown in Figure 3a (blue histogram), as an example. In order to analyze the pure glandular and adipose tissue's HU values, we applied a volumetric median filter with a kernel in the size of 3 × 3 × 3 voxels that suppresses the mixed HU signals as seen in the green histogram. A threshold (black) was applied in order to exclude the volume effects from the HU analysis of the pure adipose and glandular tissue. For the voxel value range below the threshold, these voxels were not considered in the HU analysis. Consequently, the two tissue histograms (red) were acquired.

#### 2.3.3 Standard HU measurement for each component

The mean HU value of each component was computed by the statistical analysis of the HU values from the manually segmented components in Section 2.3.1 and the HUs of the adipose and glandular tissue acquired in Section 2.3.2. The number of voxels in the statistics, parameterized mean (*μ*) and standard deviation (SD, *σ*) of the distribution, and coefficient of variation (CV, $c_{v}=\;\sigma /\left(\mu -\mathrm{Offset}\right)$) values are presented in Table 1. Due to a confidentiality reason, the mean HU values are parameterized. A schematic HU distribution of each component is demonstrated in Figure 3b. The boxes represent the IQR, and the red lines do the median in the box and whisker plots. The blue error bar represents the 95% HU range of the glandular tissue. The CV < 0.1 describes the precision of our HU measurements.

**TABLE 1:** Breast components’ Hounsfield unit (HU) calibration

Component	Counts	Mean	SD	CV
Silicone	7.0E7	$HU_{Si}$	$\sigma_{Si}$	0.05
Rib	5.7E4	$HU_{Rib}$	$\sigma_{Rib}$	0.05
Pectoralis muscle	5.6E6	$HU_{P.M.}$	$\sigma_{P.M.}$	0.06
Skin	7.3E6	$HU_{Skin}$	$\sigma_{Skin}$	0.07
Glandular tissue	1.2E8	$HU_{Gland}$	$\sigma_{Gland}$	0.02
Adipose tissue	4.7E8	$HU_{Fat}$	$\sigma_{Fat}$	0.01

Abbreviations: CV, coefficient of variation; SD, standard deviation.

### 2.4 Fully automatic volumetric segmentation and breast analysis method

The computational breast segmentation method for the spiral breast CT images was developed and applied on a computer having a central processing unit (Xeon Processor E5‐2620, Intel) and two graphics processing units (Quadro 5000, NVIDIA). The segmentation method was developed in Python programming language.

**FIGURE 4:**
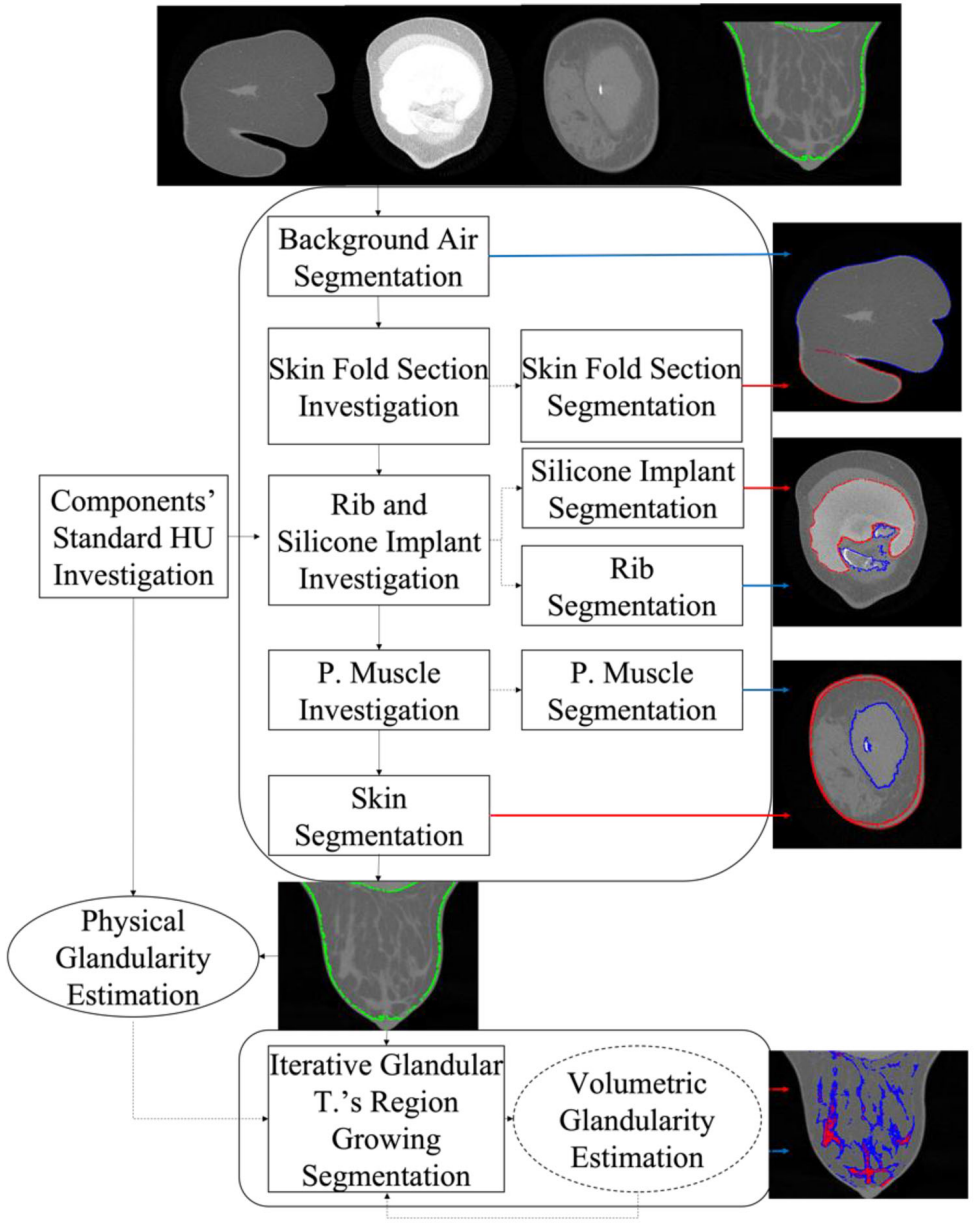
Framework of the proposed segmentation method

The sequence of our method to segment the spiral breast CT images and to estimate the breast density is shown in Figure 4. The framework is composed of four main stages as follows. In each subsequent step, the identified regions are removed from analysis except the skinfold section. The skinfold section is excluded from the glandularity analysis and segmentation of the glandular tissue, but it was included in the rest of the segmentation process. From the silicone implant to glandular tissue, the order of the subsequence follows the components’ HU values.

1. Applied to investigate the presence of the pectorals muscle, skinfold section, rib, and silicone implant and localize the seed region of each component.
2. Skin, pectoralis muscles and skinfold section, and hard components are individually segmented and separated by the breast soft tissue by applying connected component analysis (CCA),^15^ adaptive seeded watershed,^16^ and adaptive seeded region growing^17^ algorithms. These algorithms are preceded by a thresholding step with thresholds acquired from the preprocessing.
3. A preliminary estimation of the breast density is estimated.
4. The breast soft tissue that has been separated from the other components is further divided into glandular and adipose tissues.

#### 2.4.1 Investigation of presence and seed region of components

**FIGURE 5:**
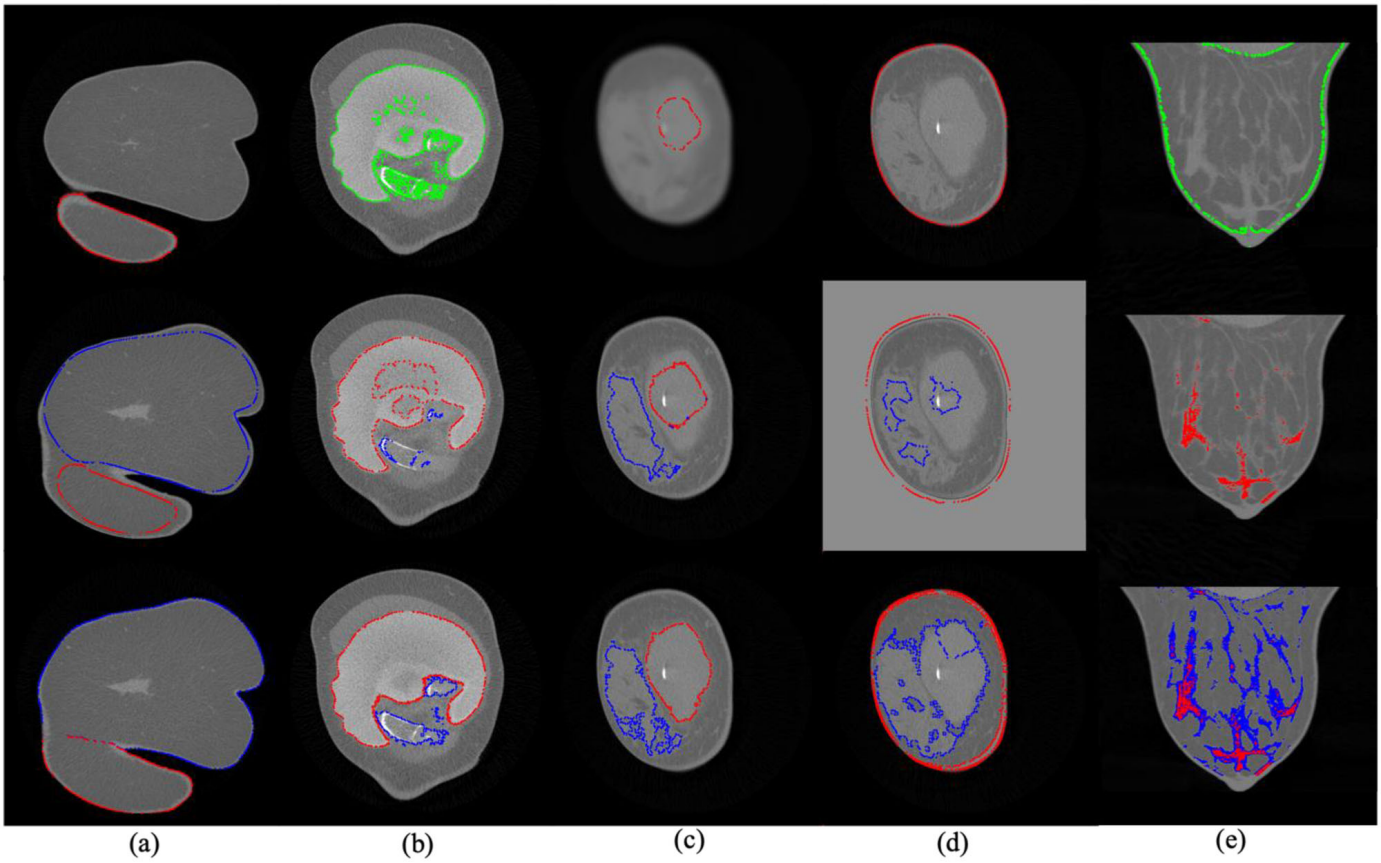
Demonstration of segmentation algorithm's process to investigate the seed region and segment the component accordingly (from top to bottom) for the skinfold section (a), silicone and rib (b), pectoralis muscle (c), skin (d), and glandular tissue (e). First row: (a), (c), and (d)—red contour represents the seed region; (b)—green contour the region to investigate the seed; (e)—green contour the confined region to segment the glandular tissue. Second row: (a), (c), and (d)—red and blue contours represent the markers to apply the adaptive watershed algorithm; (b)—red and blue contour the seeds of the respective objects; (e)—red paint the seed region. Third row: (a), (c), and (d)—red contour represents the final segmentation of the object; (b)—red and blue contours the final segmentation of the respective objects; (e)—red and blue paint together the final segmentation.

Only a part of breast CT images depicts the pectoralis muscle and non‐breast fatty fold in the images, and the shape and location vary by images. Additionally, hard materials such as the ribs and silicone implants appear in a small number of breast CT images. Our segmentation method investigated the presence of such components and localized the seed as a first step before applying the segmentation algorithms. The investigation process depends on CCA in a binary image composed of the respective component and other possible erroneous regions, which was generated by thresholding. The threshold applied to acquire the respective binary image is the mean of the reference HU of the target and surrounding components calculated in Section 2.3. The specific process for each component is described as follows:

1. Skinfold sections: The method investigates the presence of skinfolds by scanning each slice of the binary image from posterior to anterior direction applying a 2D CCA. If more than one object is detected, it labels the largest object as the seed of the breast. An exemplary breast CT image with a detected seed region of the skinfold section (red contour in the first row) is presented in Figure 5a. The seed region is expended to the volumetric region by duplicating the two 2D matrices of the skinfold seed (red) and breast (blue) toward posterior direction (the second row of Figure 5a).
2. Rib and implants: As a preprocessing, an erosion filter with a 5 × 5 × 5 kernel is applied on the respective binary image of ribs and implant. An exemplary binary image of ribs before applying the filter is marked as the green contour on top of the original breast CT image in the first row of Figure 5b. A 3D CCA is applied on the filtered binary image to mark the respective seed regions of the implant (red contour in the second row of Figure 5b) and ribs (blue contours).
3. Pectoralis muscle: In order to distinguish the muscle from the glandular tissue, a Gaussian denoise filter with a 5 × 5 × 5 kernel was applied on a breast CT image prior to the thresholding as seen in the first row of Figure 5c as an example. The largest region of the thresholded based on CCA is labeled as the seed of the muscle (red contour).

#### 2.4.2 Segmentation of components except the adipose and glandular tissues

For the strongly absorbing materials, the final contours were acquired by recovering the shape and volumes of the seed by applying morphological filters (third row of Figure 5b).

Every component of soft tissue apart from the adipose and glandular tissues is successively segmented by adaptive seeded watershed algorithms applying specifically developed markers considering the geometry and morphology of the corresponding component. A binary image applying the same threshold as (1) was generated to calculate the distance matrix for watershed algorithm. The adaptive threshold is adapted to preserve the resolution of the original image and the volume of the component. Each step of the segmentation algorithm is described as follows:

1. Skinfold sections: The two seeds of the skinfold and whole breast were adapted as the markers. By applying the adaptive seeded watershed algorithm on the distance matrix using the assigned seed and markers, the method separates the skinfold from the breast as seen in the third row of Figure 5a.
2. Pectoralis muscle: The marker was assigned as the voxels having the distance ≥upper 40% (second row of Figure 5c). The segmented muscle is presented in the third row of Figure 5c by applying the watershed algorithm with the given parameters.
3. Skin: The seed of the skin is labeled as the contour of the binary image (first row of Figure 5d). In order to distinguish the skin from the glandular tissue closely located or connected to the skin, our method adapts the binary image data in two steps: It artificially increases the distance in the skin image voxels by adding the background air voxels in the binary image; and it cuts the milk ducts that are close to the nipple and the areola area from the binary image, by applying a not operator with a sphere centered at the nipple tip with a diameter of 1.8 cm. On the adapted binary image data, the method labels the markers as the voxels having a distance larger than the median distance of all voxels (see the second row of Figure 5d). Finally, our method applies a watershed algorithm and classifies the skin after substituting the background air from and adding the cut milk ducts in the segmented object (third row of Figure 5d).
4. Adipose and glandular tissue segmentation: Adipose and glandular tissues are subsequently classified after excluding the rest of the components (first row of Figure 5e).

##### Optimization

We iteratively optimized and fine‐tuned the parameters of the algorithms and structural elements of the convolutional filters on the 68 preselected breast images for the implementation of the algorithm.

#### 2.4.3 Quantitatively estimated individual breast density

We quantitatively estimated individual's breast density based on the mean HU value of the segmented adipose and glandular tissues and the preliminarily conducted HU measurement in Section 2.3. We linearly interpolated the breast composition for the acquired mean HU of the segmented tissues with the standard HU values of the pure adipose and glandular tissues acquired in Section 2.3.2. The HU‐derived density was applied as the reference density for the glandular tissue segmentation algorithm.

#### 2.4.4 Adipose and glandular tissues’ segmentation and volumetric breast density estimation

Our adaptive region growing method is described as follows: (1) The seed of the glandular tissue was labeled by thresholding with the HU value equivalent to the pure glandular tissue (red in the second row of Figure 5e). (2) By decreasing the threshold in a step size of 2.5% of the glandular tissues’ HU value, the connected voxels in the threshold range are segmented. (3) The percentage breast density based on the two‐class segmentation matrix is estimated. (4) The error rate of the estimated volumetric segmentation‐based breast density compared to the reference is computed. (5) The algorithm iterates (2) and (3) until the absolute error rate becomes the minimum. During the iterations, the glands’ region grows to the surroundings (blue in the third row of Figure 5e) from the seed (red). Each voxel was probabilistically classified as glandular tissue with regard to the average proportion of the glandular tissue in the breast tissue volume and the relative distance to the pure glandular tissue voxel.

### 2.5 Segmentation evaluation

#### 2.5.1 Objective evaluations of automatic segmentation quality and manual segmentation reproducibility

The automatic segmentation algorithm's performance and the reproducibility in the manual segmentation were evaluated by volume overlap evaluation metrics as extensively used in the literatures^18^, ^19^: Dice's similarity coefficient (DSC)^20^ and the difference coefficient (DC) are defined as follows:

(1) $$\mathrm{DC}=\left|D_{A}\cap D_{B}\right|/\left(\left|D_{A}\left|+\right|D_{B}\right|\right)/2$$

(2) $$\mathrm{DC}=\left|D_{A}⊕D_{B}\right|/\left(\left|D_{A}\left|+\right|D_{B}\right|\right)/2$$

where, in Equations (1) and (2), *D*_A_ and *D*_B_ denote the two differently generated segmentation matrices, |*X*| denotes the cardinality of the set *X*, ∩ is the intersection of the two regions, and ⊕ is the area subtracting the intersection from the union. For our segmentation algorithm evaluation, *D*_A_ and *D*_B_ correspond to the automatically and manually segmented matrices of the object. For reproducibility assessment of the manual segmentations, *D*_A_ and *D*_B_ denote the two respective manually segmented matrices. An excellent agreement occurs when DSC > 0.75 according to previous morphological and statistical analyses.^21^, ^22^

Because DSC has a restricted range of 0,1 and is often evaluated to be close to 1 when a volume with large voxels is computed, a logit transformed DSC (LDSC) for the purpose of statistical inferences was evaluated instead:

(3) $$\mathrm{logit}\left(\mathrm{DSC}\right)=\ln(\mathrm{DSC}/(1-\mathrm{DSC}))$$

In the monotone transformation, the domain of DSC, 0,1, is mapped to the unbound range (−*∞*,*∞*). The distribution of the logit transformed proportions for a large dataset like our segmentation components (the skin, pectoralis muscle, and the skinfold), which exhibit more than 1.0E6 voxels per volume, the distributions of the resulting LDSCs follow normal distributions.^23^ Statistical tests to assess the significance in difference for normal distributed data such as Student's *t*‐test can be applied for LDSC.

Each segmentation contour by the readers A and B and our automatic method is denoted as A, B, and C, respectively. To assess the reproducibility of the manual segmentation, the DSC and DC between the A and B were computed. The comparison is denoted as AB. An example segmentation comparison is presented in the first row in Figure 2. The automatic segmentation's quality was assessed by evaluating the metrics of C (second row in Figure 2, as an example) to A and B. The comparisons are denoted as CA and CB. The DSC and DC of AB, CA, and CB were computed for the five images per each of the skin, pectoralis muscle, and skinfold. The compatibility between the reproducibility (AB) and the difference between our automatic and manual segmentations (CA and CB) were evaluated by pair‐wise *t*‐tests on the LDSC values.

#### 2.5.2 Subjective segmentation quality evaluation: Likert scale

As a commonly used metric for image quality assessment,^24^ we assessed the segmentation performance for the 25 breast CT images by a five‐point Likert scale (1 = poor, 2 = fair, 3 = moderate, 4 = good, and 5 = excellent). Five experienced radiologists having 15, 8, 8, 4, and 3 years of experience in breast imaging individually read the segmented breast CT images and categorized the segmentation quality of each following object and the overall image in comparison to the corresponding breast CT images: the fibroglandular tissue, skin, pectoralis muscle, rib, skinfold section, and silicone implant.

The interobserver agreement was assessed by computing Cronbach's alpha ($\rho_{T}$) test^25^, ^26^ for the evaluation score of each object and overall segmentation quality. Cronbach's alpha was calculated following the formula when $X_{i}$ denotes the observed score of the item *i*, *k* total item, and *X* the sum of all items in the test. $\sigma_{i}^{2}$ is the variance of $X_{i}$, and $\sigma_{X}^{2}$ consists of time variances and inter‐time covariance:

(4) $$\rho_{T}=\frac{k}{k-1}\;\left(1-\frac{\sum_{i\;=\;1}^{k}\sigma_{i}^{2}}{\sigma_{X}^{2}}\right)$$

### 2.6 Breast density estimation error assessment

The possible quantitative bias in the breast density estimation induced by the inappropriately segmented skin, pectorals muscle, and skinfold section in comparison to the true glandularity, excluding the other soft tissues, was simulated. The error rate of the breast density estimation due to the mis‐segmentation of the component was compared to the error between the estimated breast densities based on the HU value and the segmentation using our algorithm.

## 3 RESULTS

### 3.1 Evaluation of reproducibility for manual segmentations in breast CT images

The volume overlap metric analyses of the segmentations by the two readers, readers A and B, for the skin, pectoralis muscle, and skinfold were collected as seen as values of AB in Tables 2, 3, 4. The overall DSC and DC were 0.95 ± 0.02 and 0.04 ± 0.03, respectively, which represents an excellent overlap.^21^, ^22^

**TABLE 2:** Dice similarity coefficient (DSC) computation results

	AB		C(AB)	
	Result	IQR	Result	IQR
Overall	0.95 [0.91–0.98]	0.04	0.94 [0.86–1.00]	0.10
Skin	0.94 [0.91–0.97]	0.04	0.89 [0.86–0.91]	0.00
Pectoralis muscle	0.94 [0.91–0.96]	0.05	0.94 [0.88–0.99]	0.04
Skinfold section	0.96 [0.94–0.98]	0.03	0.99 [0.97–1.00]	0.00

**TABLE 3:** Difference coefficient (DC) computation results

	AB		C(AB)	
	Result	IQR	Result	IQR
Overall	0.04 [0.00–0.14]	0.06	0.06 [0.00–0.13]	0.09
Skin	0.05 [0.04–0.07]	0.03	0.09 [0.06–0.12]	0.05
Pectoralis muscle	0.08 [0.01–0.14]	0.10	0.07 [0.00–0.02]	0.08
Skinfold section	0.00 [0.00–0.01]	0.00	0.01 [0.00–0.02]	0.01

**TABLE 4:** Logit transformed Dice similarity coefficient (LDSC) computation results

	AB		C(AB)	
	Result	IQR	Result	IQR
Overall	2.99 [2.25–4.13]	0.74	3.25 [1.84–5.32]	2.37
Skin	2.85 [2.25–3.37]	0.72	2.09 [1.84–2.36]	0.04
Pectoralis muscle	2.78 [2.33–3.28]	0.85	2.88 [2.02–3.58]	0.75
Skinfold section	3.34 [2.79–4.13]	0.75	4.79 [4.39–5.32]	0.56

### 3.2 Evaluation of breast component's presence investigation

**FIGURE 6:**
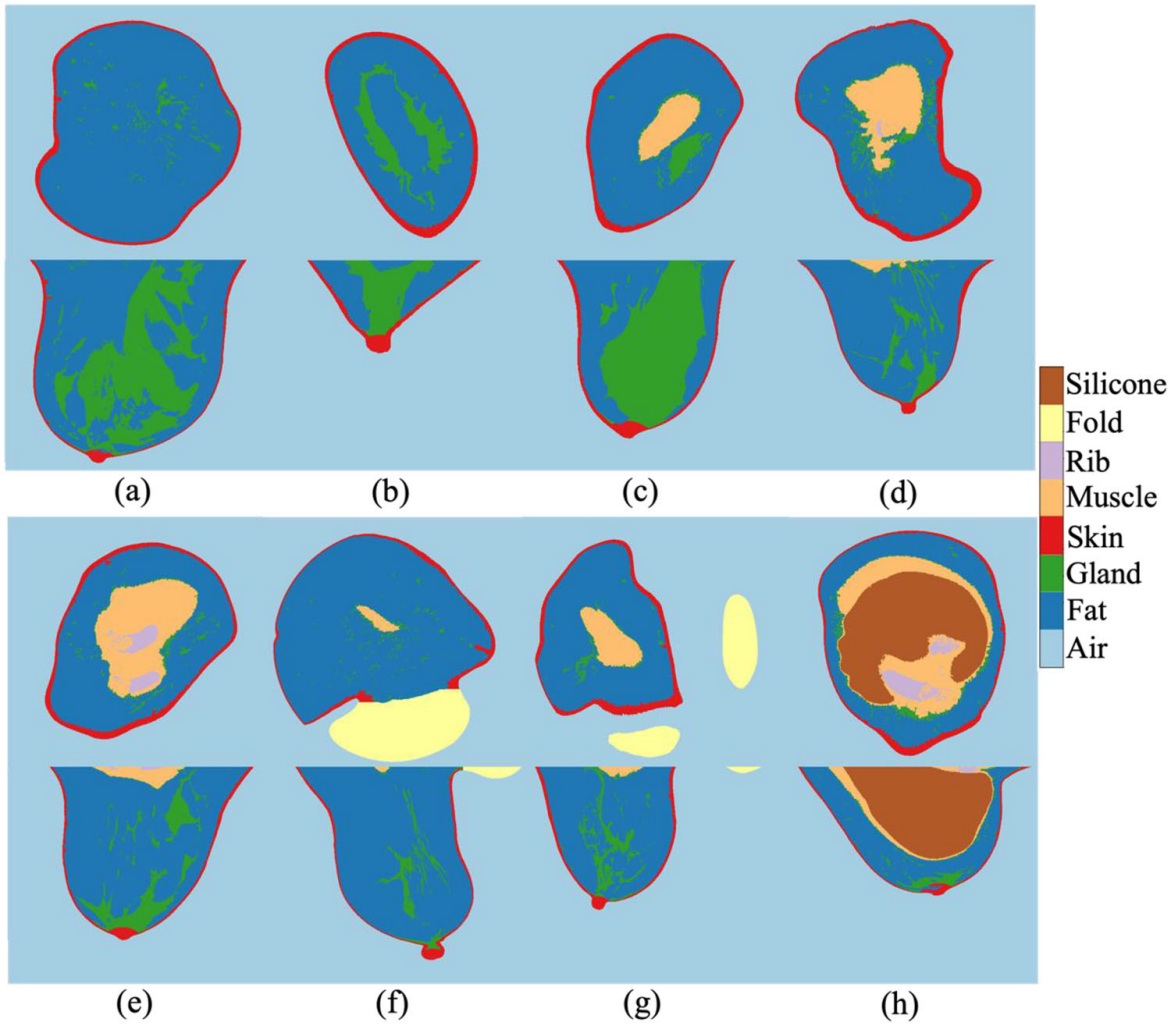
Segmented breast computed tomography (CT) images corresponding to the respective examplary images [a‐h] in Figure 1

Among the images in the evaluation set successfully segmented by our method, the segmentation result of the eight representative patient cases in Figure 1 is presented in Figure 6. Our segmentation method depicted every component that additionally appears in the breast CT images besides the glandular and adipose tissues and skin: the pectoralis muscle, rib, skinfold, and silicone implant. Our CCA was able to investigate ambiguous cases: non‐breast skinfolds attached to the breast (e.g., Figures 2c and 6f); multiple ribs besides the silicone implant (Figure 6h); and pectoralis muscle that appears with a compact gland mass (Figures 2a,b and 6c). The denoise preprocessing step allowed the CCA to distinguish the compact glandular tissues from the pectoralis muscle.

### 3.3 Evaluation of strongly absorbing component segmentation

**FIGURE 7:**
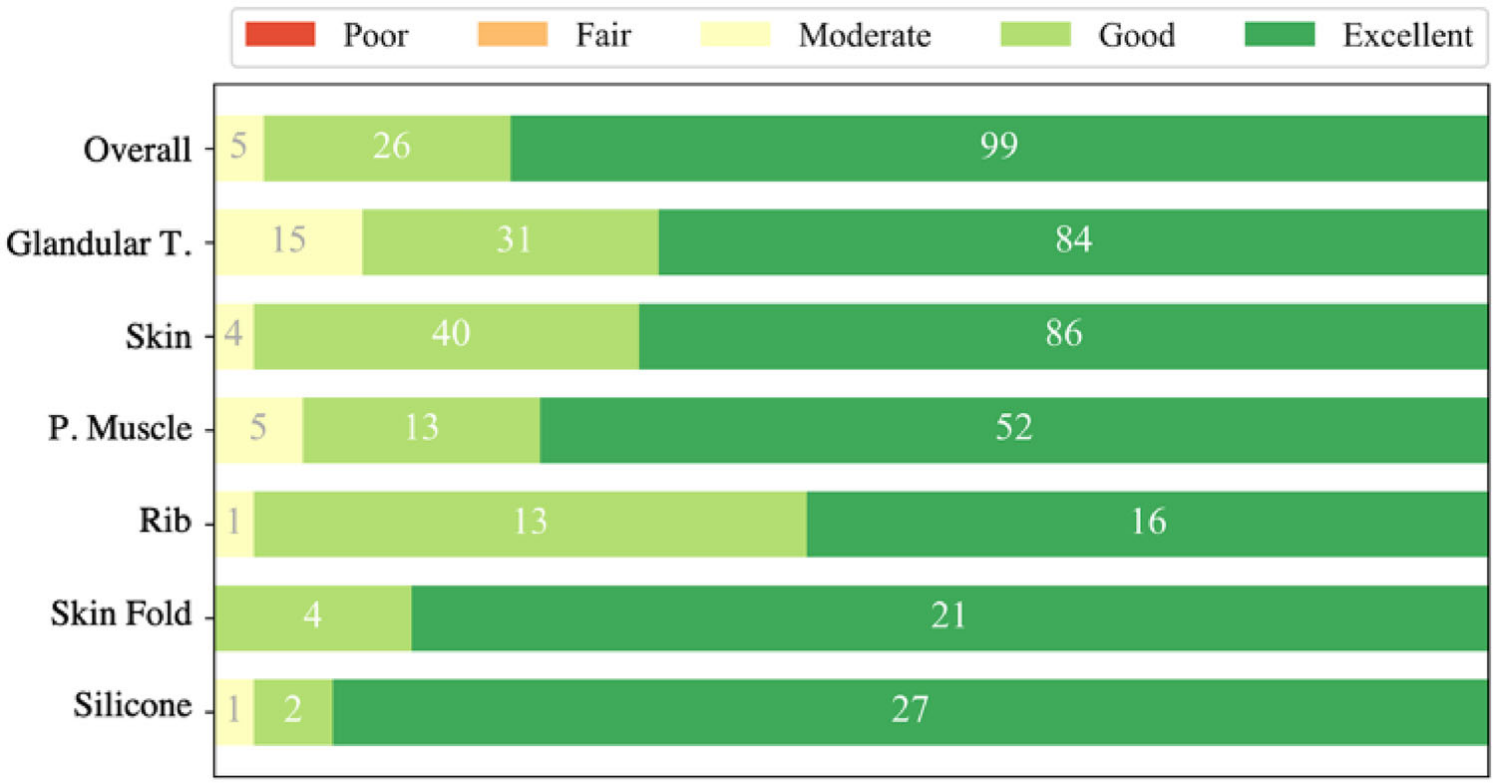
Subjective evaluation result in five‐point Likert scale

The radiologists evaluated the segmentation of the ribs and silicone implants as 4.8 ± 0.4 and 4.9 ± 0.4, respectively, in a five‐point Likert scale. The assessment result in five‐point Likert scale for each component's segmentation is presented in Figure 7. Cronbach's alpha coefficients among the evaluations by the five raters for 550 objects were 0.83, which infers that the inter‐reader reliability in the evaluation was excellent ($\rho_{T}$ > 0.8).

### 3.4 Evaluation of soft tissue component segmentation by adaptive seeded watershed algorithm

The histogram‐based markers allowed accurate segmentation of the skin and pectoralis muscle in close contact to the fibroglandular tissue. The skin was segmented with the original thickness, which differs in each breast and even substantially varies within the same breast. The pectoralis muscles having various structures from a typical round shape with clear border (Figure 6e,f) to a scattered shape with irregular border (Figure 6d) or a structure connected to the glandular tissue (Figures 2a,b and 6c,g) were precisely segmented. The method generated an agreeable separation of the skinfold from the breast when they are attached (Figures 2c and 6f). In images having a minor deformation such as a partial moving artifact, our segmentation method performed without a major malfunction in the segmentation process or inadequate segmentation.

Our computational segmentation method showed an excellent overlap with the reference segmentation by the human readers.^21^, ^22^ The DSC and DC analysis on the skin, pectoralis muscle, and skinfold segmentation (CA and CB combined) resulted in 0.94 ± 0.02 and 0.05 ± 0.05, respectively (C(AB) in Tables 2, 3, 4). The volumetric overlaps between the automatic and the manual segmentations (CA and CB) and between the manual segmentations (AB) were not significantly different. The paired *t*‐tests on the LDSC values resulted in 0.452 and 0.926 for the comparison between CA and AB and between CB and AB, respectively. The null hypothesis that the LDSC of the paired sets come from normal distributions with equal mean was not rejected. The subjective evaluation by the human raters also resulted as good to excellent for the three soft tissue components. The score in the five‐point Likert scale was 4.6 ± 0.5 for skin, 4.7 ± 0.6 for pectoralis muscle, and 4.8 ± 0.4 for skinfold section.

### 3.5 Evaluation of the glandular tissue segmentation: adaptive region growing algorithm

Our adaptive region growing algorithm successfully segmented the glandular tissue while conserving the quantitative volumetric segmentation‐based density close to the density of the reference evaluation. In the histogram‐based probabilistic segmentation adapting the information of the fibroglandular structure, the partial volume effect and system blurring was well addressed, and the ring and radial line artifacts were excluded. The segmentation quality on the glandular tissue was rated as 4.5 ± 0.7 in five‐point Likert scale by the five experts (Figure 7).

### 3.6 Quantitative breast density estimation

**FIGURE 8:**
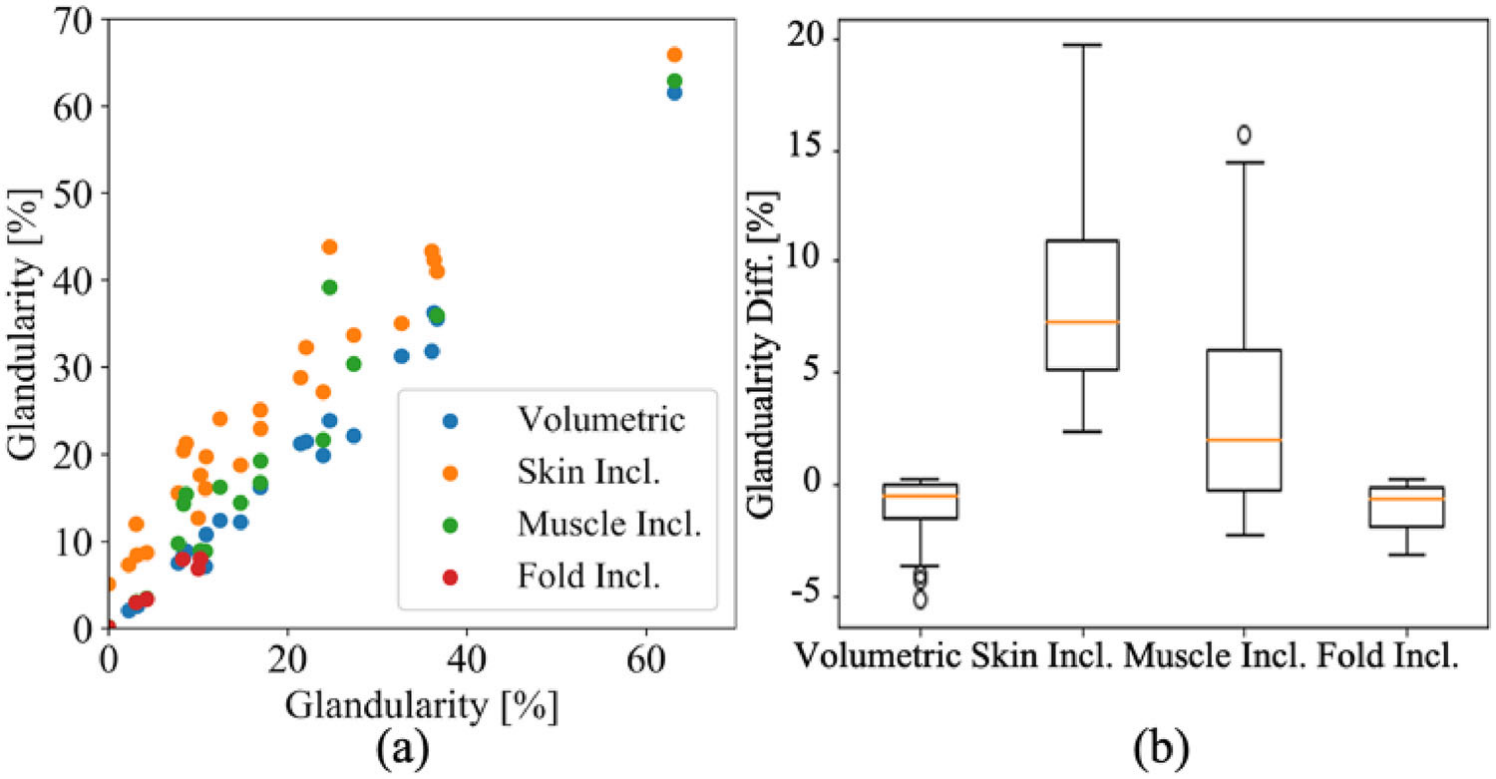
Glandularity plot (a) and box‐and‐whisker diagram (b) of the estimated glandularity differences compared to the correct Hounsfield unit (HU)‐derived estimation and the volumetric segmentation‐based estimation or the erroneous HU‐derived estimation with the mis‐segmented skin, pectoralis muscle, or skinfold section.

Four variables of the breast density estimated by the segmentation method are plotted in Figure 8a as a function of the HU‐derived breast density acquired in Section 2.4.3: (1) without any mis‐segmentation, including (2) the skin, (3) pectoralis muscle, or (4) skinfold section as the glandular tissue. The results of the statistical analysis on corresponding estimation differences to the HU‐derived values are presented in Table 5 and a box‐and‐whisker diagram, as shown in Figure 8b. The error rates between the volumetric segmentation‐based and HU‐derived breast density estimations were 1.2% ± 1.6% in average. The skin and pectoralis muscle inclusion in the breast density estimation induced 7.1% ± 3.7% and 3.7% ± 4.7% of significant bias, respectively (*p* < 0.05), whereas the skinfold inclusion did 0.4% ± 0.2% in the segmentation‐based breast density.

**TABLE 5:** Estimated glandularity differences between the correct Hounsfield unit (HU)‐derived estimation and the volumetric segmentation‐based estimation or the erroneous HU‐derived estimation with the mis‐segmented skin, pectoralis muscle, or skinfold section (%)

	Mean [Min–Max]	IQR
Volumetric	−1.2 [−5.2−0.0]	2.03
Skin incl.	7.1 [2.3–19.1]	4.38
Muscle incl.	3.7 [0.0–18.1]	5.41
Fold incl.	0.4 [0.1–0.5]	0.26

### 3.7 Processing time of the computational and manual segmentations for breast CT images

The segmentation method took in average 12.8 ± 10.1 min of computational time per a spiral breast CT image when no silicone implant is present and 20.9 ± 5.5 min when an implant is included in the dataset. The radiologists took about 250 min per breast. The operation time in each component's segmentation by the automatic and manual segmentation is presented in Table 6.

**TABLE 6:** Computation time

		Total	Gland	Skin	Muscle	Rib	Skinfold	Silicone
Without silicone	Mean	12.8	3.0	8.3	2.1	0.2	0.3	–
	SD	10.1	6.9	3.9	0.8	0.1	0.2	–
With silicone	Mean	20.9	1.3	7.8	10.7	0.3	–	1.1
	SD	5.5	0.7	1.7	5.0	–	–	0.2

Abbreviation: SD, standard deviation.

## 4 DISCUSSION

We propose here for the first time a fully automated segmentation method that can be applied to individual spiral breast CT datasets. The presented method is a combination of deterministic and probabilistic model image processing and analysis techniques. The method consists of four main steps: investigation of the breast components and localization of the seeds; segmentation of the breast component apart from the adipose and glandular tissues; quantitative breast density estimation; and glandular tissue segmentation. The qualities of automatic segmentations of breast components were evaluated by five experienced radiologists in a five‐point Likert scale with inter‐reader reliability test based on Cronbach's alpha test. The segmentation of the skin, pectoralis muscle, and skinfolds were compared to manual segmentations by two radiologists by computing DSC and DC. Overall, qualitative and quantitative results show that the obtained segmentations were equivalent to the reference standard of human reading: 4.7 ± 0.5 (good–excellent) in a five‐point Likert scale with an excellent inter‐reader agreement ($\rho_{T}$ > 0.8) and 0.94 ± 0.04 in DSC evaluation. The DSCs of the skin, pectoralis muscle, and non‐breast fatty folds were 0.89 ± 0.08, 0.94 ± 0.04, and 0.99 ± 0.01, respectively. In the skin, pectoralis muscle, and skinfold segmentation, the automatic and manual segmentations were not significantly different from the reproducibility between two manual segmentations as seen in the *t*‐test results on LDSC values.

The method automatically localized the seed of the skin and each additional component by applying the preprocessing, CCA, despite the image noise and overlapping HU distributions between the soft tissues. During the preprocessing, our method did not require parabolic correction or image normalization that involves additional uncertainties in the gray level in the segmentation thanks to the reliable HU values, unlike the method requiring such preprocessing steps for cone‐beam breast CT (CBCT)^27^ and MR^7^, ^8^, ^9^, ^18^, ^28^ images. Thanks to the robustness of the adaptive seeded watershed algorithms, our method segmented the skin and pectoralis muscle adapting inter‐ and intra‐patient variability in shape for individual patient despite the surrounding or physically connected glandular tissue. A new adaptive region growing algorithm classified the glandular tissue by applying the average breast density for the voxel‐wise probability. In the glandular tissue segmentation, purely histogram‐based probabilistic methods, such as the expectation maximization (EM) algorithm, were excluded in our segmentation method in order to take the relative location to the seed gland voxel and the macroscopic glandularity into consideration.

One of the main contributions of this paper is to compute the quantitative and reader‐independent density for the first time from the HU values of the breast image excluding the skin, pectoralis, and non‐breast fatty fold. We demonstrated the erroneously segmented skin or pectoralis muscle could cause a significant bias in the breast density estimation by artificially increasing the density. Mis‐segmentation of the skin and pectoralis muscle induced 7.1% ± 3.7% and 3.7% ± 4.7% bias in average and 19.1% and 18.1% in maximum, respectively, in the quantitative breast glandularity estimation. For a further breast density estimation study on a large patient cohort, appropriate segmentation of the pectoralis muscle that appears in 57% of breast CT scans^29^ is necessary to avoid 3.7% ± 4.7% uncertainty.

Various breast segmentation methods have been implemented for MRIs and CBCT. The whole breast volume segmentation on MRIs resulted in the DSC in the range of 0.93–0.96 by applying an edge extraction filter combined with the candidate evaluation,^18^ probabilistic atlas method,^8^ depth field modeling with self‐adaptation features,^28^ or CNN model, U‐net.^9^ The DSC in comparison with an expert‐outlined reference for the CWL (pectoralis muscle for spiral breast CT) by Gubern‐Merida's method using an atlas‐based approach resulted in 0.75 ± 0.09.^8^, ^30^ Caballo,^31^ Yang,^32^ and Packard^27^ introduced a glandular tissue segmentation method on CBCT image using an unsupervised algorithm, modified fuzzy *C*‐mean classification, and two mean clustering. Caballo's method resulted in DSC of 0.90–0.95. For breast MRI, Wu,^7^ Gubern‐Merida,^8^ and Dalmış^9^ proposed the glandular tissue segmentation method by applying the atlas‐aided fuzzy *C*‐mean method, the EM, and U‐net, respectively. The DSC of the fibrogland segmentation resulted for Wu's method in the range of 0.61–0.69,^21^, ^22^ and for Gubern‐Merida and Dalmış’s in the range of 0.80–0.85, respectively.

As Refs.^9^, ^31^ proposed to apply a CNN method for the whole breast segmentation in MR image, the use of a CNN is a recent trend that has shown success in image analysis. The time required to manually generate the segmented datasets for the complex fibroglands in the high‐resolution datasets of 684 × 684 × (600–1000) voxels, however, is still a limiting factor of this technique. Our adaptive region growing algorithm's computation time was 3.0 ± 6.9 min per breast, which is substantially faster than other methods that take similar time for MRI images of a relatively smaller data size.^7^, ^8^, ^9^

Our study has several limitations. First, the number of test image datasets evaluated was limited. In order to cope with the limitation and reduce the potential bias, the selection of test dataset included a variety of breast structure, and an equal number of imager sets were selected from each of the four BI‐RADS density classifications. Second, due to the design of the segmentation workflow, the deterministic method can be sensitive to major breast deformations and cannot distinguish pathological lesions in the breast during the segmentation. In the case of a partly resected breast, for example, erroneous glandular tissue segmentation may occur when the scar from surgery extends deeply into the breast. A pathological mass or microcalcifications presented in the image may be classified as glandular tissue or skin. The limiting factors of our method could overcome with integration with a CNN. Third, there is an uncertainty in the HU analysis for the adipose and glandular tissue, because a median filter tends to bias histograms toward the middle. The bias due to the application of a median filter might need to be further investigated.

The proposed segmentation method can in principle be applied as a standalone tool, for example, to provide the description of individual breast tissue structures and complement existing software in the clinical workflow. The current processing time for a complete breast segmentation ranges between 10 and 15 min and, therefore, is compatible with the typical duration of an examination. Using the relevant components’ localization and size assessment, the CT image acquisition quality and the quantitative breast density can be assessed on site. Another potential application of our automatized breast segmentation lies in further studies, such as the epidemiological assessment of breast densities and the analysis of the effective dose based on the segmented breast images for a large cohort. Our method provides the geometrical distribution of glandular tissue and skin that substantially affects the effective organ dose due to the concave radiation distribution^14^, ^33^ and enables a reliable effective dose analysis.

We proposed a fully automated segmentation method for spiral breast CT. The method segmented adipose and glandular tissue, skin, pectoralis muscle, ribs, skinfold section depicted from abdominal or thoracic wall, and silicone implant and estimate the quantitative breast density. The automatic segmentation results coincided well with the human expert's reading. The result of the segmentation and breast density estimation demonstrated that an accurate segmentation is important to avoid a significant bias in breast density analysis. Our method enabled accurate quantification of the breast density and amount of the glandular tissue that is directly related to breast cancer risk.

## CONFLICT OF INTEREST

All authors declare that they have no conflicts of interest.

## AUTHOR CONTRIBUTION

All authors contributed significantly to the performed work and approved to the final version of the manuscript to be published.

## Data Availability

Research data are not shared due to privacy or ethical restrictions.
